# Psychological distress and quality of life in breast cancer survivors with taxane-induced peripheral neuropathy: A scoping review

**DOI:** 10.3389/fonc.2022.1005083

**Published:** 2023-01-10

**Authors:** Lauren Schwab, Constance Visovsky

**Affiliations:** College of Nursing, University of South Florida, Tampa, FL, United States

**Keywords:** breast cancer survivors, psychological distress, chemotherapy induced peripheral neuropathy, quality of life, taxane

## Abstract

**Purpose:**

This review provides an assessment of psychological distress (depressive symptoms and anxiety symptoms) and the impact on quality of life in breast cancer survivors with chemotherapy-induced peripheral neuropathy induced by taxane-based chemotherapy.

**Methods:**

The databases PubMed, CINAHL, Embase, and PsychInfo were searched for publications about psychological distress symptoms in breast cancer survivors with chemotherapy-induced peripheral neuropathy from taxane chemotherapy and the impact on quality of life.

**Results:**

Only eight studies were identified that addressed psychological distress symptoms in BCS with CIPN treated with taxane chemotherapy and the impact of these symptoms on QOL. Of these studies, a majority reported increased symptoms of psychological distress (depressive symptoms and/or anxiety symptoms) in BCS with CIPN. Researchers found that the persistent nature of CIPN and severity of symptoms resulted in decreased QOL.

**Conclusions:**

This review highlighted a notable lack of research on psychological distress (depressive symptoms and anxiety symptoms) in BCS with CIPN. Furthermore, there was a gap in knowledge in how this psychological distress impacts QOL in this population. Further research is needed to better understand the extent that BCS with CIPN experience symptoms of psychological distress and the impact on QOL. This research would enable researchers to develop interventions focused on decreasing and potentially preventing these symptoms in BCS with CIPN treated with taxane chemotherapy, thereby decreasing the impact on QOL.

## Introduction

Breast cancer is the most common cancer in women in the United States (U.S.) and an estimated 1 in 8 women (13%) will receive a breast cancer diagnosis in her lifetime (1). In 2022, in the U.S., it is estimated that approximately 287,850 women will receive a new diagnosis of invasive breast cancer (1). In 2021, worldwide, breast cancer became the most commonly occurring cancer accounting for approximately 12% of all newly diagnosed cancers (2). Increased awareness, earlier screening and detection, and advancements in treatments have led to over 4.1 million breast cancer survivors (BCS) living in the U.S. (1). Concurrent to the increase in survivorship, many BCS experience adverse effects from chemotherapy treatment for months to years after initial diagnosis and last chemotherapy cycle (3–6). Taxanes [paclitaxel (Taxol) and docetaxel (Taxotere)] are a commonly prescribed chemotherapeutic agent used to treat both invasive and metastatic breast cancer, which can have a direct effect on both the central and peripheral nervous systems (3, 7–9). Although taxanes dramatically improve the 5-year survival rate and decrease the risk of recurrence, there is an increased risk of both long-term physical [e.g., chemotherapy-induced peripheral neuropathy (CIPN)] and psychological (e.g., depressive symptoms and anxiety symptoms) side effects, which can negatively impact quality of life (QOL) (3, 6, 7, 10, 11).

CIPN occurs in approximately 80-97% of patients treated with taxane chemotherapy (12–14). Taxane-induced CIPN is a chronic, debilitating, and distressing complication that presents in a stocking-and-glove distribution that manifests as symptoms of paresthesia, numbness, burning, pain, altered temperature perception (e.g., cold allodynia or increased sensitivity to cold), myalgia, myopathy, difficulty with fine motor skills, impaired gait and balance, muscle weakness in the lower extremities, and/or functional decline (10, 12–15). Many patients with taxane-induced CIPN experience persistent symptoms, which may promote and/or exacerbate symptoms of psychological distress (depressive symptoms and anxiety symptoms) and result in decreased QOL (3, 11).

## Psychological distress

Although the concept of “psychological distress” has not reached consensus, the majority of researchers agree that the phrase may be used to describe the multifactorial experience of psychological symptoms which may range from symptoms of sadness and fear to more debilitating symptoms of depression and anxiety, which may interfere with daily functioning and significantly impact QOL (16). For the purposes of this review, psychological distress will be used to describe depressive and anxiety symptoms.

Depression and anxiety are relatively common conditions in the general population with reports suggesting an estimated 8.4% of all adults in the U.S. have had at least one major depressive episode and up to 19.1% have had an anxiety disorder within the past year (17, 18). However, compared to the general population, psychological distress is much more prevalent in BCS with some reports suggesting that the prevalence of depression and anxiety symptoms among BCS may be as high as 66.1% and 33.3%, respectively (19).

## Methods

### Search strategy

A literature search was performed using the databases PubMed, CINAHL, Embase, and PsychInfo. Specific search terms used were “depression” OR “anxiety” OR “quality of life” OR “mental health” OR “emotional health” OR “emotional distress” AND “breast cancer” OR “breast neoplasm” OR “breast carcinoma” OR “breast tumor” AND “chemotherapy induced peripheral neuropathy” OR “CIPN”. A complex search strategy was used combining key words and was guided by a medical librarian. The MeSH (Medical Subject Headings) terms that were used in the search were “depression”, “anxiety”, “quality of life”, and “breast neoplasms”. Of the articles included in the review, full reference lists were reviewed for additional relevant articles.

### Inclusion and exclusion criteria

Inclusion criteria comprised research articles published between 2012-2022 in the English language that included an assessment of psychological distress (depressive symptoms and anxiety symptoms) and the impact on QOL in female BCS with CIPN from taxane chemotherapy. This timeframe was chosen to ensure the most current and comprehensive results were included. Articles were excluded if CIPN was not specific to breast cancer; articles focused on CIPN in BCS, but did not address or measure symptoms of psychological distress (depressive symptoms and/or anxiety symptoms) and/or QOL. Articles were also excluded if they included patients currently undergoing treatment.

### Search outcome

The initial search produced 441 results. A total of 163 duplicates were removed; a further 9 articles were removed for not meeting publication date (n=6) and language criteria (n=3). A total of 269 articles remained, which were screened by title and abstract. An additional 221 articles were excluded for not meeting the inclusion criteria. This resulted in a total of 48 articles for full-text review. After reviewing the remaining articles, 8 articles met all inclusion criteria and were included in the review (see **Appendix A**).

### Data analysis

This scoping review was guided by the framework created by Arksey and O’Malley (20), which was further revised by Levac and colleagues in 2010. Arksey and O’Malley (20) assert that one of the main reasons for conducting a scoping review is to identify gaps in the existing literature about a certain topic, which was the primary objective for this review. The goal of a scoping review and how it differs from a systematic review is to identify all relevant literature about a topic including both qualitative and quantitative research (20). Arksey and O’Malley’s (20) framework consists of five stages: 1) Identifying the research question; 2) identifying relevant studies; 3) study selection; 4) charting the data; and 5) collating, summarizing, and reporting results. This scoping review was conducted to answer the research question: What is known from the existing literature about psychological distress (depressive symptoms and anxiety symptoms) in BCS with taxane-induced peripheral neuropathy and the impact on QOL? This research question was used to identify and select relevant studies for this review. After all relevant studies were selected and reviewed for inclusion in this review, data were charted into a table format (see **Appendix B**). Following charting of the data from the included studies, results could be collated, summarized, and reported in a narrative format.

## Results

Publication dates for included articles ranged from 2015-2022. Study designs of the included studies varied. Two of the articles were cross-sectional (7, 10), five were prospective (21–25), and one was longitudinal (3).

One of the included studies was from the U.S.A. (10), three were from Denmark (3, 21, 24), one was from the Netherlands (25), one was from Portugal (23), and two were from South Korea (7, 22). All studies reported a sample with a similar mean age (ranging between 44.2 and 62.5 years). The two studies from South Korea by Kim and Jung (7) and Lee etal. (22) had on average a lower mean age, 49.9 and 44.2, respectively. The mean age of these studies coincides with the most common age group for breast cancer occurrence in South Korea, the 40-49 age group (26).

### Sample characteristics

Three of the studies included participants with stage I, II, and III breast cancer (3, 7, 10). One study only included participants with stage II and III breast cancer (22) and another study included participants with “high risk” breast cancer (21). The article by Bennedsgaard etal. (21) included two groups of participants, one with breast cancer treated with docetaxel and one with colorectal cancer treated with oxaliplatin, and results of this study were distinguishable by cancer type. In two studies, participants only received treatment with taxane-based chemotherapy (10, 21). Two studies included participants who received a combination of anthracyclines and cyclophosphamides followed by docetaxel (3, 22). Kim and Jung (7) included participants who received taxane-containing and non-taxane containing chemotherapy.

### Instruments measuring psychological distress

Only one measure of psychological distress (depressive and anxiety symptoms) was consistent: the Hospital Anxiety and Depression Scale (HADS). This measure is a self-report questionnaire that was developed for use in the outpatient setting for identifying depression and anxiety states (27). The HADS may also be used for assessing severity of anxiety and depression disorders (27). The HADS has been used extensively in cancer patients and was shown to be effective in identifying anxiety and depressive disorders in this population, which makes it a suitable choice for assessing psychological distress in breast cancer patients (28). Five of the included studies used the HADS for assessing psychological distress (depressive symptoms and anxiety symptoms) in BCS with CIPN treated with taxane chemotherapy (10, 21–24). Other researchers used the Patient Health Questionnaire9 (PHQ-9) to measure depressive symptoms (7, 25), the Brief Symptom Inventory 18 (BSI-18) to measure psychological distress (7), and the Generalized Anxiety Disorder-7 to measure anxiety symptoms (25).

### Quality of life instruments

The European Organization for the Research and Treatment of Cancer Quality of Life Questionnaire (EORTC QLQ-C30) was the most frequently used measures to assess QOL in BCS with CIPN. The EORTC QLQ-C30 is composed of five scales for assessing a patient’s functioning (physical, emotional, social, cognitive, and role), eight symptom scales, global QOL scale, and is one of the most widely used measurement tools for assessing QOL in cancer research (29). Other measurement tools that were used for assessing QOL in this population were the EuroQol-5 Dimension (EQ-5D) and the EORTC QLQ-CIPN20.

### Psychological distress in BCS with taxane-induced CIPN

Most of the included studies reported some form and/or level of psychological distress (depressive symptoms and anxiety symptoms) among patients experiencing symptoms of CIPN following taxane chemotherapy. However, since each of the studies used different methods and instruments for measuring psychological distress and QOL, comparing the results from the included studies was difficult. Three of the eight included studies reported an association between CIPN symptoms and depression symptoms (7, 10, 21). Participants treated with taxane who developed CIPN following treatment reported increased depressive symptomology (10). Furthermore, CIPN severity was closely related to greater levels of depression (7). Bennedsgaard and colleagues (21) found that when compared to other breast cancer treatments, those patients treated with taxanes experienced greater psychological distress manifested by depressive symptoms.

Six of the eight included studies reported an association between CIPN and anxiety (7, 10, 21, 22, 24, 25). Increased anxiety levels were significantly associated with CIPN symptom severity (7). Interestingly, two of the included studies reported that pre-treatment anxiety was associated with the development and persistence of CIPN following taxane chemotherapy (22, 25).

### Impact of CIPN on QOL

Four of the included studies reported on QOL measures related to CIPN. Eckhoff etal. (3) found that 15% of BCS who were treated with taxane-based chemotherapy experienced persistent CIPN, which had a negative effect on QOL. Another study also found that treatment with taxanes resulted in persistent CIPN, 5 years after treatment, which lead to a decreased QOL (21). In contrast, Pereira and colleagues (23) reported that CIPN in BCS did not have a significant impact on QOL at 1-year follow-up, which the authors related to decreased severity of CIPN symptoms, as well as improved symptoms over this time period.

## Discussion

The cancer experience alone can lead to psychological distress (depression symptoms and anxiety symptoms) in BCS, however when accompanied by a persistent, debilitating comorbidity, such as CIPN, these symptoms of psychological distress may worsen and negatively impact QOL (30). Furthermore, in BCS with CIPN, symptoms of psychological distress can increase the severity and perception of CIPN symptoms (16). On the other hand, the symptom experience of CIPN following taxane chemotherapy can increase the severity of psychological distress symptoms (depression symptoms and anxiety symptoms) (16). CIPN following taxane-based chemotherapy in BCS are known side effects of chemotherapy, however there is a paucity of research on the interrelationship between psychological distress (depression and anxiety symptoms) and their impact on QOL in this population.

Symptoms of psychological distress (depression and anxiety symptoms) often persist for years after the last taxane chemotherapy cycle and can have a major impact on QOL in BCS with CIPN (31). QOL is a complex, multidimensional concept that is affected by both physical and psychological functioning (32, 33). After treatment, many BCS continue to experience persistent symptoms of psychological distress, which may be related to a number of factors, such as fear of recurrence, post-traumatic stress, or chronic, physical side effects (34). Mental health issues in BCS are a prominent concern and it is imperative that they receive as much attention as physical side effects from cancer treatment because they can significantly impact QOL. Furthermore, BCS have an increased risk for long-term psychological distress, which highlights the importance of screening and identifying these symptoms so that appropriate referrals can be made and their psychological needs are met (34, 35).

Potential strategies for increasing QOL in BCS include maintaining a healthy weight, regular physical activity and exercise, healthy diet (e.g., Mediterranean diet) and nutrition, abstaining from alcohol, sun protection, and regular follow-up with health care providers (36–38). Obesity and lack of physical activity are highly related to cancer recurrence (39). Exercise improves physical and mental health and ultimately increases QOL for BCS (37, 40–43). Additionally, in BCS exercise has been shown to reduce fatigue, depression, anxiety, and stress (44).

## Strengths and limitations

To the best of the author’s knowledge, this is the first review to examine psychological distress manifested by anxiety and depression symptoms, experienced by BCS with CIPN treated with taxane chemotherapy. The strengths of this review are the systematic and thorough methodology used as guided by Arksey and O’Malley’s (20) framework for scoping reviews. However, some limitations must be acknowledged. First, only studies published in English were included in this review, therefore, relevant articles published in other languages may have been missed. Second, comparing psychological distress and CIPN between studies was problematic due to the variety of measurement tools that were used in the included studies. Also, there was a lack of consistency in follow-up time and type of chemotherapy regimen, which also made comparison difficult. A limitation of the use of a scoping review is that this methodology does not incorporate a quality assessment, however lack of consensus about whether quality assessment is necessary for this type of review.

## Conclusion

This review has highlighted a significant gap in the research regarding psychological distress experienced by BCS with CIPN. More research is needed to address the psychological concerns of this population to decrease symptoms of psychological distress (depression and anxiety symptoms). An additional topic of concern is establishing a gold standard diagnostic tool for identifying anxiety and depression in cancer survivors to ensure that they are receiving appropriate support and referrals. Psychological well-being is just as important as physical well-being and needs to be prioritized for cancer survivors by both researchers and health care providers as both can have a significant negative impact on QOL. Additional research is needed to identify ways to mitigate psychological distress symptoms in BCS with CIPN.

## Author contributions

LS contributed substantially to the conception and design of the manuscript and wrote the first draft, CV organized the manuscript, wrote sections of the manuscript, and revised it critically for intellectual content. All authors contributed to the manuscript, read, and approved the submitted version of the manuscript.
