# Supplementary material for: Psychological distress and quality of life in breast cancer survivors with taxane-induced peripheral neuropathy: A scoping review
Source: Front Oncol. 2023 Jan 10;12:1005083. doi: 10.3389/fonc.2022.1005083 (PMC9872004; doi:10.3389/fonc.2022.1005083)
Supplement: Supplementary file 1 [file DataSheet_1.docx]

**Appendix A**

*PRISMA Flow Diagram*

**Identification of studies via databases and registers**

Studies included in review (n = 8)

**Included**

Reports excluded:

Articles including patients currently undergoing treatment (n=37)

Articles including no measures of psychological distress or quality of life (n=3)

Reports assessed for eligibility (n = 48)

Records screened by title and abstract (n=269)

Records removed before screening: (n=172)

Duplicate records removed (n=163)

Records remoed for other reasons (n=9)

More than 10 years (n=6)

Not in English (n=3)

Records excluded for not meeting inclusion criteria (n=221)

Records identified from databases (n=441):

CINAHL (n=48)

Embase (n=265)

PsychInfo (n=6)

PubMed (n=74)

**Screening**

**Identification**
