# Supplementary material for: Psychological distress and quality of life in breast cancer survivors with taxane-induced peripheral neuropathy: A scoping review
Source: Front Oncol. 2023 Jan 10;12:1005083. doi: 10.3389/fonc.2022.1005083 (PMC9872004; doi:10.3389/fonc.2022.1005083)
Supplement: Supplementary file 2 [file DataSheet_2.docx]

**Appendix B**

*Table 1. Study Characteristics and Outcomes*

| **Study (Country)** | **Purpose** | **Design** | **Sample Characteristics** | **Results** | **Reported Measures** |
| --- | --- | --- | --- | --- | --- |
| Bao et al., 2016,  (USA) | To determine CIPN prevalence, RFs, and association with psychological distress and falls among long-term BCS | Cross-sectional | - N = 296 post-menopausal women with stage I-III breast cancer who received taxane chemotherapy - Mean age = 62.0 (SD = 9.0) | 173 (58.4%) reported CIPN symptoms, of these 91 (30.7%) had mild CIPN and 82 (27.7%) had moderate to severe CIPN.  Additionally, obesity was associated with an increased risk of CIPN (adjusted OR 1.9, 95%).  Patients with CIPN had greater insomnia, anxiety, and depression (p<.05).  More severe CIPN was associated with higher rate of falls (p=.028) | Psychological Distress: HADS |
| Bennedsgaard et al., 2020,  (Denmark) | To assess CIPN 5 year after adjuvant chemo in pts with breast and CRC and to analyze association of CIPN with QOL, anxiety, and depression. | Long-term, prospective | - N = 80 patients with breast cancer who received taxane chemotherapy - Mean age at 5 year follow-up: 56.6 | 80% (38.8%) had numbness and tingling and neuropathic pain in the feet (p<.001).  Patients with symptoms of neuropathy (tingling and numbness) had significantly lower QOL scores than those without neuropathy (p=.011).  Those treated with taxanes experienced greater anxiety and depression symptoms vs. those only treated with surgery  Symptoms of CIPN after taxanes persisted 5 years after treatment ended and negatively impacted QOL resulting in lower QOL | Psychological Distress: HADS  QOL: EQ-5D |
| Eckhoff et al., 2015, (Denmark) | To assess duration and severity of PN after cessation of docetaxel by analyzing PROs in a longitudinal study and to evaluate the impact of persistence of PN on HRQOL | Longitudinal | - N = 1031 patients with early-stage breast cancer who received at least one cycle of docetaxel - Median age: 52 | 241 (23%) patients reported CIPN grades 2-4. Persisted 1-3 years among 81 patients (34%).  Persistent CIPN had a significant negative correlation with HRQOL, function scales, and symptom scales (p<.0001). | Psychological Distress and QOL: EORTC QLQ-C30 |
| Kim & Jung, 2021, (South Korea) | To examine how influencing factors (physiologic, psychological, and situational) affect CIPN symptoms and the impact of symptom experience on functional interference in daily activities of chemotherapy-treated BCS. | Cross sectional descriptive testing hypothetical SEM | - N = 190 women treated with adjuvant chemotherapy for non-metastatic breast cancer - Mean age: 49.91 | Physiologic factors including breast cancer stage, cumulative taxane dose, length of time since chemotherapy completion contribute to CIPN (p<.05).  Psychologic factors such as anxiety and depression are related to CIPN symptoms (p<.001). | Psychological Distress: PHQ-9, BSI-18 |
| Lee et al., 2018,  (South Korea) | To explore incidence and RDFs of persistent CIPN among women with breast cancer receiving neoadjuvant chemotherapy and to identify these RFs, including psychiatric factors that contribute to development and persistence of CIPN. | Prospective | - N = 111 women with breast cancer receiving neoadjuvant chemotherapy including 4 cycles of docetaxel - Mean age: 44.17 | 50 (45%) had CIPN during chemotherapy, 21 (18.9%) had persistent CIPN after chemotherapy.  Development of ([OR] 4.02; 95% CI, p=.033) and persistent (p=0.32) CIPN was significantly associated with pre-treatment numbness and pre-treatment anxiety. Pre-treatment anxiety was significantly associated with persistent CIPN (p=.020). | Psychological Distress: HADS |
| Pereira et al., 2016,  (Portugal) | To estimate incidence of CIPN and to identify its main determinants and impact in patient-reported outcomes | Prospective cohort | - N = 296 patients with breast cancer who had chemotherapy and were followed for 1 year - <50 years = 46% - $\geq50$= 54% | Incidence of CIPN in the first year was 28.7% (95% CI).  80% of patients had symptomatic CIPN at 6 months.  The risk for CIPN was higher for treatment with docetaxel.  Did not find significant impact on QOL and patient-reported outcomes at 1-year follow-up, which authors relate to lower severity of taxane-induced CIPN. | Psychological Distress: HADS  QOL: EORTC QLQ-C30 |
| Ventzel et al., 2016  (Denmark) | To prospectively compare and characterize development of CIPN symptoms and pain sensory profiles and also psychological functioning in pts treated with either adjuvant docetaxel or oxaliplatin, and to identify predictors of chronic CIPN and CIPN-related pain. | Prospective | - N = 276 - n = 100 in docetaxel group (breast cancer group) - Mean age of docetaxel group = 51.6 | 44.8% in the docetaxel group had chronic CIPN symptoms at 1-year follow-up.  Persistent pain in patients receiving docetaxel was found to have an effect on psychological function.  In the HADS, at the 1-year follow-up there were significant differences in anxiety and depression (p<.05). | Psychological Distress: HADS  QOL: EQ-5D |
| Verhoeff-Jahja et al., 2022  (Netherlands) | To examine the relationship between symptoms of anxiety and depression before the start of chemotherapy and development of CIPN 6 months after completing treatment in women with breast cancer. | Prospective | - N = 61 women with breast cancer receiving neoadjuvant taxane chemotherapy - Mean age = 51.7 | Patients with medium/high levels of anxiety at baseline showed a significantly higher increase in CIPN symptoms during and after chemotherapy than those women with low levels at baseline (p<.001). | Psychological Distress: GAD-7, PHQ-9  QOL: EORTC QLQ-CIPN20 |
