# Supplementary material for: Psychological distress and quality of life in breast cancer survivors with taxane-induced peripheral neuropathy: A scoping review
Source: Front Oncol. 2023 Jan 10;12:1005083. doi: 10.3389/fonc.2022.1005083 (PMC9872004; doi:10.3389/fonc.2022.1005083)
Supplement: Supplementary file 3 [file DataSheet_3.docx]

**References**

Aaronson, N. K., Ahmedzai, S., Bergman, B., Bullinger, M., Cull, A., Duez, N. J., Filiberti, A., Flechtner, H., Fleishman, S. B., & de Haes, J. C. (1993). The European Organization for Research and Treatment of Cancer QLQ-C30: A quality-of-life instrument for use in international clinical trials in oncology. *Journal of the National Cancer Institute*, *85*(5), 365–376. https://doi.org/10.1093/jnci/85.5.365

Annunziata, M. A., Muzzatti, B., Bidoli, E., Flaiban, C., Bomben, F., Piccinin, M., Gipponi, K. M., Mariutti, G., Busato, S., & Mella, S. (2020). Hospital Anxiety and Depression Scale (HADS) accuracy in cancer patients. *Supportive Care in Cancer*, *28*(8), 3921–3926. https://doi.org/10.1007/s00520-019-05244-8

Argyriou, A. A., Kyritsis, A. P., Makatsoris, T., & Kalofonos, H. P. (2014). Chemotherapy induced peripheral neuropathy in adults: a comprehensive update of the literature. *Cancer management and research*, *6*, 135–147. https://doi.org/10.2147/CMAR.S44261

Arksey, H. & O’Malley, L. (2005). Scoping studies: Towards a methodological framework. *International Journal of Social Research Methodology, 8*(1). http://doi.org/10.1080/1364557032000119616

Bao, T., Basal, C., Seluzicki, C., Li, S. Q., Seidman, A. D., & Mao, J. J. (2016). Long-term chemotherapy-induced peripheral neuropathy among breast cancer survivors: Prevalence, risk factors, and fall risk. *Breast Cancer Research and Treatment*, *159*(2), 327–333. https://doi.org/10.1007/s10549-016-3939-0

Bennedsgaard, K., Ventzel, L., Themistocleous, A. C., Bennett, D. L., Jensen, A. B., Jensen, A. R., Andersen, N. T., Jensen, T. S., Tankisi, H., & Finnerup, N. B. (2020). Long-term symptoms of polyneuropathy in breast and colorectal cancer patients treated with and without adjuvant chemotherapy. *Cancer medicine*, *9*(14), 5114–5123.

https://doi-org.ezproxy.hsc.usf.edu/10.1002/cam4.3129

Brown, T. J., Sedhom, R., & Gupta, A. (2019). Chemotherapy-induced peripheral neuropathy. *JAMA Oncology*, *5*(5), 750. https://doi.org/10.1001/jamaoncol.2018.6771

Centers for Disease Control and Prevention (CDC). (2018). HRQOL concepts. http://www.cdc.gov/hrqol/concept.htm

Duijts, S. F., Faber, M. M., Oldenburg, H. S., van Beurden, M., & Aaronson, N. K. (2011). Effectiveness of behavioral techniques and physical exercise on psychosocial functioning and health-related quality of life in breast cancer patients and survivors--a meta analysis. *Psycho-oncology*, *20*(2), 115–126. https://doi.org/10.1002/pon.1728

Eckhoff, L., Knoop, A., Jensen, M. B., & Ewertz, M. (2015). Persistence of docetaxel-induced neuropathy and impact on quality of life among breast cancer survivors. *Eur J Cancer, 51*(3), 292-300. http://doi.org/10.1016/j.ejca.2014.11.024

Giaquinto, A. N., Sung, H., Miller, K. D., Kramer, J. L., Newman, L. A., Minihan, A., Jemal, A., & Siegel, R. L. (2022). Breast cancer statistics, 2022. *CA A Cancer J Clin.* http://doi.org/10.3322/caac.21754

Haidinger, R., & Bauerfeind, I. (2019). Long-term side effects of adjuvant therapy in primary breast cancer patients: Results of a web-based survey. *Breast Care (Basel, Switzerland)*, *14*(2), 111–116. https://doi.org/10.1159/000497233

Hashemi, S. M., Rafiemanesh, H., Aghamohammadi, T., Badakhsh, M., Amirshahi, M., Sari, M., Behnamfar, N., & Roudini, K. (2020). Prevalence of anxiety among breast cancer patients: A systematic review and meta-analysis. *Breast cancer (Tokyo, Japan)*, *27*(2), 166–178. https://doi.org/10.1007/s12282-019-01031-9

Hong, J. S., Tian, J., & Wu, L. H. (2014). The influence of chemotherapy-induced neurotoxicity on psychological distress and sleep disturbance in cancer patients. *Current Oncology (Toronto, Ont.)*, *21*(4), 174–180. https://doi.org/10.3747/co.21.1984

Hwang, E. S., & Nho, J. H. (2019). Lifestyle Intervention for Breast Cancer Women. *Journal of Lifestyle Medicine*, *9*(1), 12–14. https://doi.org/10.15280/jlm.2019.9.1.12

Kang, S. Y., Kim, Y. S., Kim, Z., Kim, H. Y., Kim, H. J., Park, S., Bae, S. Y., Yoon, K. H., Lee, S. B., Lee, S. K., Jung, K. W., Han, J., Youn, H. J., & Korean Breast Cancer Society. (2020). Breast cancer statistics in Korea in 2017: Data from a breast cancer registry. *Journal of breast cancer*, *23*(2), 115–128. https://doi.org/10.4048/jbc.2020.23.e24

Kerckhove, N., Collin, A., Condé, S., Chaleteix, C., Pezet, D., & Balayssac, D. (2017). Long term effects, pathophysiological mechanisms, and risk factors of chemotherapy-induced peripheral neuropathies: A comprehensive literature review. *Frontiers in pharmacology*, *8*, 86. https://doi.org/10.3389/fphar.2017.00086

Kim, M., & Jung, M. S. (2021). Effects of chemotherapy-induced peripheral neuropathy in women with breast cancer: A structural equation approach with the theory of unpleasant symptoms. *Cancer Nursing*, *44*(2), 145–153. https://doi.org/10.1097/NCC.0000000000000764

Lauby-Secretan, B., Scoccianti, C., Loomis, D., Grosse, Y., Bianchini, F., Straif, K., & International Agency for Research on Cancer Handbook Working Group (2016). Body fatness and cancer--viewpoint of the IARC Working Group. *The New England Journal of Medicine*, *375*(8), 794–798. https://doi.org/10.1056/NEJMsr1606602

Lee, K. M., Jung, D., Hwang, H., Son, K. L., Kim, T. Y., Im, S. A., Lee, K. H., & Hahm, B. J. (2018). Pre-treatment anxiety is associated with persistent chemotherapy-induced peripheral neuropathy in women treated with neoadjuvant chemotherapy for breast cancer. *Journal of psychosomatic research*, *108*, 14–19.

https://doi-org.ezproxy.hsc.usf.edu/10.1016/j.jpsychores.2018.02.012

Levkovich, I., Cohen, M., Alon, S., Kuchuk, I., Nissenbaum, B., Evron, E., Pollack, S., & Fried, G. (2018). Symptom cluster of emotional distress, fatigue and cognitive difficulties among young and older breast cancer survivors: The mediating role of subjective stress. *Journal of Geriatric Oncology, 9*(5), 469-475. http://doi.org/10.1016/j.jgo.2018.05.002

Lovelace, D. L., McDaniel, L. R., & Golden, D. (2019). Long-term effects of breast cancer surgery, treatment, and survivor care. *Journal of Midwifery & Women’s Health, 64*(6), 713-724. http://doi.org/10.1111/jmwh.13012

Maass, S. W., Roorda, C., Berendsen, A. J., Verhaak, P. F., & de Bock, G. H. (2015). The prevalence of long-term symptoms of depression and anxiety after breast cancer treatment: A systematic review. *Maturitas*, *82*(1), 100–108.

https://doi-org.ezproxy.hsc.usf.edu/10.1016/j.maturitas.2015.04.010

Miaskowski, C., Mastick, J., Paul, S. M., Topp, K., Smoot, B., Abrams, G., Chen, L., Kober, K. M., Conley, Y. P., Chesney, M., Bolla, K., Mausisa, G., Mazor, M., Wong, M., Schumacher, M., & Levine, J. D. (2017). Chemotherapy-induced neuropathy in cancer survivors. *Journal of Pain and Symptom Management, 54*(2), 204-218e2. http://dx.doi.org/10.1016/j.jpainsymman.2016.12.342

Montagnese, C., Porciello, G., Vitale, S., Palumbo, E., Crispo, A., Grimaldi, M., Calabrese, I., Pica, R., Prete, M., Falzone, L., Libra, M., Cubisino, S., Poletto, L., Martinuzzo, V., Coluccia, S., Esindi, N., Nocerino, F., Minopoli, A., Grilli, B., Fiorillo, P. C., … Augustin, L. (2020). Quality of life in women diagnosed with breast cancer after a 12 month treatment of lifestyle modifications. *Nutrients*, *13*(1), 136. https://doi.org/10.3390/nu13010136

National Cancer Institute (NCI). (2021). PDQ supportive and palliative care editorial board: PDQ adjustment to cancer. *National Cancer Institute.*

https://www.cancer.gov/about-cancer/coping/feelings/anxiety-distress-hp-pdq

National Institute of Mental Health (NIMH). (n.d.) Any anxiety disorder. https://www.nimh.nih.gov/health/statistics/any-anxiety-disorder#part_2579

National Survey of Drug Use and Health (NSDUH) (2022). 2020 National Survey of Drug Use and Health (NSDUH) release. *Substance Abuse and Mental Health Services Administration (SAMHSA).* https://www.samhsa.gov/data/sites/default/files/reports/slides-2020-nsduh/2020NSDUH NationalSlides072522.pdf

Paek, M. S., Wong, S. S., Hsu, F. C., Avis, N. E., Fino, N. F., & Clark, C. J. (2021). Depressive symptoms and associated health-related variables in older adult breast cancer survivors and non-cancer controls. *Oncology nursing forum*, *48*(4), 412–422. https://doi.org/10.1188/21.ONF.412-422

Patsou, E. D., Alexias, G. T., Anagnostopoulos, F. G., & Karamouzis, M. V. (2018). Physical activity and sociodemographic variables related to global health, quality of life, and psychological factors in breast cancer survivors. *Psychology research and behavior management*, *11*, 371–381. https://doi.org/10.2147/PRBM.S170027

Pereira, S., Fontes, F., Sonin, T., Dias, T., Fragoso, M., Castro-Lopes, J. M., & Lunet, N. (2016). Chemotherapy-induced peripheral neuropathy after neoadjuvant or adjuvant treatment of breast cancer: a prospective cohort study. *Supportive care in cancer : official journal of the Multinational Association of Supportive Care in Cancer*, *24*(4), 1571–1581. https://doi.org/10.1007/s00520-015-2935-y

Pinto, B. M., Dunsiger, S., & Waldemore, M. (2013). Physical activity and psychosocial benefits among breast cancer patients. *Psycho-oncology*, *22*(10), 2193–2199. https://doi.org/10.1002/pon.3272

Porciello, G., Montagnese, C., Crispo, A., Grimaldi, M., Libra, M., Vitale, S., Palumbo, E., Pica, R., Calabrese, I., Cubisino, S., Falzone, L., Poletto, L., Martinuzzo, V., Prete, M., Esindi, N., Thomas, G., Cianniello, D., Pinto, M., Laurentiis, M., Pacilio, C., … Augustin, L. (2020). Mediterranean diet and quality of life in women treated for breast cancer: A baseline analysis of DEDiCa multicentre trial. *PloS One*, *15*(10), e0239803. https://doi.org/10.1371/journal.pone.0239803

Rogers, L. Q., Courneya, K. S., Anton, P. M., Hopkins-Price, P., Verhulst, S., Vicari, S. K., Robbs, R. S., Mocharnuk, R., & McAuley, E. (2015). Effects of the BEAT Cancer physical activity behavior change intervention on physical activity, aerobic fitness, and quality of life in breast cancer survivors: a multicenter randomized controlled trial. *Breast cancer research and treatment*, *149*(1), 109–119.

https://doi.org/10.1007/s10549-014-3216-z

Schmidt, M. E., Wiskemann, J., & Steindorf, K. (2018). Quality of life, problems, and needs of disease-free breast cancer survivors 5 years after diagnosis. *Quality of Life Research, 27*, 2077-2086. http://doi.org/10.1007/s11136-018-1866-8

Seretny, M., Currie, G. L., Sena, E. S., Ramnarine, S., Grant, R., MacLeod, M. R., Colvin, L. A., & Fallon, M. (2014). Incidence, prevalence, and predictors of chemotherapy-induced peripheral neuropathy: A systematic review and meta-analysis. *Pain, 155*(12), 246- 2470. http://doi.org/10.1016/j.pain.2014.09.020

Sung, H, Ferlay, J,  Siegel, RL,  Laversanne, M,  Soerjomataram, I,  Jemal, A, & Bray, F. (2021). Global cancer statistics 2020: GLOBOCAN estimates of incidence and mortality worldwide for 36 cancers in 185 countries. *CA Cancer Clin, 71*(3), 209-249. http://doi.org/10.3322/caac.21660

The World Health Organization Quality of Life Assessment (WHOQOL): Development and general psychometric properties. (1998). *Social science & medicine (1982)*, *46*(12), 1569–1585. https://doi.org/10.1016/s0277-9536(98)00009-4

Ventzel, L., Jensen, A. B., Jensen, A. R., Jensen, T. S., & Finnerup, N. B. (2016). Chemotherapy-induced pain and neuropathy: A prospective study in patients treated with adjuvant oxaliplatin or docetaxel. *Pain*, *157*(3), 560–568. https://doi.org/10.1097/j.pain.0000000000000404

Verhoeff-Jahja, R., Ter Kuile, M. M., Weijl, N. I., Oosterkamp, R., Cloos, M., Portielje, J., Kroep, J. R., & Hinnen, C. (2022). Symptoms of anxiety but not depression before start of taxane-based chemotherapy are associated with peripheral neuropathy: A multicenter study in women with breast cancer. *Supportive Care in Cancer*, *30*(8), 6947–6953. https://doi.org/10.1007/s00520-022-07093-4

Willson, M. L., Burke, L., Ferguson, T., Ghersi, D., Nowak, A. K., & Wilcken, N. (2019). Taxanes for adjuvant treatment of early breast cancer. *The Cochrane Database of Systematic Reviews*, *9*(9), CD004421. https://doi.org/10.1002/14651858.CD004421.pub3

Yang, T.-Y., Chen, M.-L. and Li, C.-C. (2015), Effects of an aerobic exercise programme on fatigue for patients with breast cancer undergoing radiotherapy. J Clin Nurs, 24: 202- 211. https://doi.org/10.1111/jocn.12672

Yi, J. C., & Syrjala, K. L. (2017). Anxiety and depression in cancer survivors. *The Medical Clinics of North America*, *101*(6), 1099–1113. https://doi.org/10.1016/j.mcna.2017.06.005

Zigmond, A. S., & Snaith, R. P. (1983). The hospital anxiety and depression scale. *Acta psychiatrica Scandinavica*, *67*(6), 361–370. https://doi.org/10.1111/j.16000447.1983.tb09716.x
